# Concordance of Hypermethylated DNA and the Tumor Markers CA 15-3, CEA, and TPA in Serum during Monitoring of Patients with Advanced Breast Cancer

**DOI:** 10.1155/2015/986024

**Published:** 2015-08-03

**Authors:** Søren Kristiansen, Lars Mønster Jørgensen, Morten Høgh Hansen, Dorte Nielsen, György Sölétormos

**Affiliations:** ^1^Department of Clinical Biochemistry, North Zealand Hospital, University of Copenhagen, Dyrehavevej 29, 3400 Hillerød, Denmark; ^2^Department of Oncology, Herlev Hospital, University of Copenhagen, Herlev Ringvej 75, 2730 Herlev, Denmark

## Abstract

The serological protein tumor markers CA 15-3, CEA, and TPA are frequently used to monitor tumor burden among metastatic breast cancer patients. Breast cancer is associated with global DNA hypomethylation and hypermethylation of some promoter regions. No monitoring study has yet investigated the interrelationship between protein tumor markers, the global DNA hypomethylation, and hypermethylated genes in serum from patients with advanced disease. Twenty-nine patients with histologically proven advanced breast cancer received first-line chemotherapy with epirubicin. Samples were collected prior to each treatment and prospectively analyzed for CA 15-3, CEA, and TPA. The same samples were retrospectively analyzed for the concentration of hypermethylated *RASSF1A* and for global DNA hypomethylation using *LINE-1*. Among patients with elevated concentrations of the protein markers, concordance could be observed between serial changes of the hypermethylated *RASSF1A* gene and the protein markers. Among patients with lower concentrations, *RASSF1A* could only be detected periodically. There was discordance between changes of the hypomethylated *LINE-1* as compared to the protein markers. Circulating hypermethylated *RASSF1A* and protein markers may have similar kinetics during monitoring of tumor burden. Further investigations are needed to determine whether any of the hypermethylated DNA genes may provide predictive information during monitoring.

## 1. Introduction

Monitoring the treatment of metastatic breast cancer involves a wide array of assessments and the need for the clinician to integrate several different forms of information about the effectiveness of treatment and the acceptability of toxicity [1]. The information includes those from direct observations of the patient including patient reported symptoms; performance status; change in weight; physical examination; laboratory tests such as alkaline phosphatase, liver function, blood counts, and calcium; radiographic imaging; functional imaging; and, where appropriate, tumor biomarkers [1].

The tumor markers cancer antigen 15-3 (CA 15-3), carcinoembryonic antigen (CEA), and tissue polypeptide antigen (TPA**)** can optionally be used as a supplement to monitor the effect of the anticancer treatment in metastatic breast cancer [1]. The CA 15-3 assay is based on the monoclonal antibodies 115D8 and DF3 which are both raised against the human MUC1 protein [2, 3]. The CEA molecule is a glycoprotein involved in cell adhesion. CEA is a glycosylphosphatidylinositol cell surface anchored glycoprotein that is released into the bloodstream of cancer patients and healthy individuals [2, 3]. Being a secretory product both CA 15-3 and CEA are considered as serological markers of changing tumor burden in the individual patient; however, the exact mechanism of release from the cell membrane is unknown. TPA belongs to the cytoskeleton proteins circulating as a complex of soluble proteolytic polypeptide fragments of cytokeratins 8, 18, and 19 [2, 3]. Their release may indicate cell turnover and the information supplied by TPA may be distinctly different from the information supplied by the markers of tumor burden CA 15-3 and CEA [2, 3].

The results of all clinical evaluations, that is, physical evaluations, laboratory tests, imaging, and serum biomarkers, generally are classified as response, continued response to treatment, stable disease, uncertainty regarding disease status, or progression of disease [1]. The clinicians typically must assess and balance multiple different forms of information to make a determination regarding whether disease is being controlled and the toxicity of treatment is acceptable [1]. Sometimes this information may be contradictory, and recent guidelines do not recommend the use of serum tumor biomarkers alone in metastatic breast monitoring for evaluating the response to anticancer therapy [1]. The average sensitivity for CA 15-3, CEA, and TPA is 70%, 55%, and 64%, respectively, for breast cancer at stage IV, and it drops to 35%, 25%, and 40% at stage III [2].

The significance of hypermethylation of tumor suppressor genes in carcinogenesis is being increasingly recognized as new serum biomarkers for monitoring metastatic breast cancer [5–7]. Some of the potential interesting hypermethylated genes associated with breast cancer have been reviewed recently [6–8]. Interestingly, the hypermethylated RAS association (RalGDS/AF-6) domain family member 1A gene (*RASSF1A*) has been reported to have clinical sensitivity of 67%–75% for stage IV breast cancer [9]. Lastly, the long interspersed nuclear elements (*LINE*) are a member of the autonomous retrotransposons encoding for a reverse transcriptase and are transcribed by a RNA polymerase II. This transposable element can change its position within the genome, and the* LINE-1* gene is one of the most abundant sequences in the human genome and makes up 17% of the human genome.* LINE-1* is often used as a surrogate for global hypomethylation, and quantification of* LINE1* in circulating DNA is suggested as a molecular biomarker of breast cancer [10]. Thus, hypermethylated* RASSF1A* and hypomethylation of* LINE-1* are candidates for clinical research studies of novel serological biomarkers for monitoring breast cancer.

So far, no studies have compared the kinetics of hyper- and hypomethylated DNA with the kinetics of CA 15-3, CEA, and TPA protein tumor markers during monitoring of advanced breast cancer [1]. In the present study, we have monitored the serial changes in the hypermethylated* RASSF1A* and global hypomethylation using* LINE-1* and compared with the changes in CA 15-3, CEA, and TPA concentrations.

## 2. Materials and Methods 

### 2.1. Healthy Subjects

Women among the healthy staff at the Departments of Oncology and Clinical Chemistry, Her1ev Hospital, University of Copenhagen, Denmark, volunteered to participate in the study from 1990 to 1992 [11]. All subjects gave informed consent to their participation, and the study was approved by the regional Ethical Committee (KA 93076). All subjects stated they were free of disease at the time of the study, and none had any known chronic or recurrent illness or was taking any medication. The subjects continued their usual lifestyle during the period of the study. No investigations were performed to exclude asymptomatic breast cancer. Serum samples were stored at −80°C and later analyzed for hypermethylated* RASSF1A*.

### 2.2. Patients with Advanced Breast Cancer

The 29 investigated patients had histologically proven advanced progressive breast cancer with measurable or evaluable disease [4, 12]. They received epirubicin 70 mg/m^2^ on days 1 and 8 every 4 weeks. Epirubicin was continued until progressive disease (PD) was noted or until a maximum cumulative dose of 1000 mg/m^2^ had been administered. Pretreatment evaluation includes a complete history and physical examination, blood cell counts (hemoglobin, WBC, and platelets), serum chemistry profiles (creatinine, calcium, alkaline phosphatase, transaminase, and bilirubin), chest radiography, electrocardiography, ^51^Cr-EDTA clearance, and bone scans. Areas of increased uptake on bone scans were further evaluated with roentgenograms to determine the nature of the abnormalities. Ultrasound scan of the liver was performed if the serum alkaline phosphatase or transaminase was elevated [12]. During treatment, history taking, physical examination, blood cell counts (hemoglobin content, leukocytes, and platelets), and routine biochemistry (sodium, potassium, creatinine, calcium, magnesium, alkaline phosphatase, aspartate aminotransferase, lactate dehydrogenase, and bilirubin) were repeated before each treatment cycle. Evaluable or measurable indicators were evaluated every second month, except for bone lesions, which were evaluated every third month [4]. The clinical study were carried out in the period from 1988–1991. In that period the clinical response evaluations were based on the criteria of the World Health Organization [13]. Clinical response evaluation was performed by investigators without knowledge of the tumor marker data. Blood specimens for CA 15-3, CEA, and TPA analysis were sampled before each treatment cycle [4, 12]. Each specimen was analyzed for CA 15-3, CEA, and TPA. The specimens were analyzed consecutively, and each specimen from an individual patient was analyzed in a separate assay run. Changes in marker concentrations were evaluated by criteria as described by Sölétormos et al. [4]. Additional specimens were sampled whenever data for alkaline phosphatase, aspartate aminotransferase, lactate dehydrogenase, or calcium were requested outside the scheduled time points. The CA 15-3, CEA, and TPA concentrations were assessed by one investigator who had not participated in the clinical evaluation. At each sampling the serum specimen used for analysis of the protein tumor markers was saved in different aliquots at −80°C and used for the current analysis of hyper- and hypomethylated DNA. The study complied with the Helsinki II Declaration and was approved by the Scientific Ethics Committee of Copenhagen County (KA 89257, H-D-2009-048).

Serum DNA was isolated using the High Pure Viral Nucleic Acid kit (Roche Diagnostics, Mannheim, Germany) and subjected to sodium bisulfite conversion of nonmethylated cytosines (EpiTect Bisulfite kit from QIAGEN) and stored at −80°C. The probe and primer designs used for the hypermethylated* RASSF1A* and collagen 2 gene (*COL2A1*) have previously been reported [14, 15]. The primers targeted non-CpG-containing regions of* COL2A1*. The measured* COL2A1* concentration is therefore not sensitive to any potential methylation of CpG dinucleotide motifs and can therefore be used to measure the assay input of DNA. The* COL2A1* concentration was used to normalize the* RASSF1A* concentration. The PCR reaction was carried out with the 7500 Fast Real-Time PCR system (Applied Biosystem) using the TaqMan Genotyping Master mixture. The analytical coefficient of variance for the detection of hypermethylated* RASSF1A* gene was 10.9%.

The* LINE-1* gene was used as a surrogate for global hypomethylation by analyzing the concentration of methylated and unmethylated* LINE-1* by methylation-specific PCR [16]. A standard curve was prepared by using bisulfite-converted DNA from MCF7 breast cancer cells. The* LINE-1* amplicons were investigated by melt curve analysis and UV illumination of ethidium stained amplicons separated on 2% (wt./vol.) 1x TBE agarose gels. The percentage of methylated* LINE-1* was calculated using the formula: 100 × methylated reaction/(unmethylated reaction + methylated reaction). Relative %* LINE-1* methylation was investigated in serial samples obtained from six of the patients (total 71 serial serum samples). The analytical coefficient of variance for the* LINE-1* methylation-specific PCR method on 7500 Fast Real-Time system was 15.6%.

## 3. Results

Hypermethylated* RASSF1A* was not detected in serum samples obtained from eighteen healthy women with a mean age of 62.8 years (range 55–75). Thus, the clinical specificity of hypermethylated* RASSF1A* was 100%. The mean age of the twenty-nine patients was 49.6 years (range 34–67 year), and the mean length of the individual therapy period was 196 days (range 59–396 days) consisting of a mean number of 6.5 cycles per patient. The distribution of metastasis before start of therapy and at the end of therapy is shown in Table 1. The patients had metastasis at multiple locations (22 out of 29 patients), in the lymph node (14 out of 29) and bones (13 out of 29) before start of therapy. After the end of therapy, the majority of patients still had metastasis at multiple locations, bones, lymph nodes, lung, liver, and other sites. When comparing the status of metastasis before therapy with the status after therapy, there was a reduction in number of patients with metastasis in the lymph nodes, lung, skin, solitary locations, and multiple locations and an increase in number of patients with metastasis in the liver, bone, and other locations.

**Figure 1:**
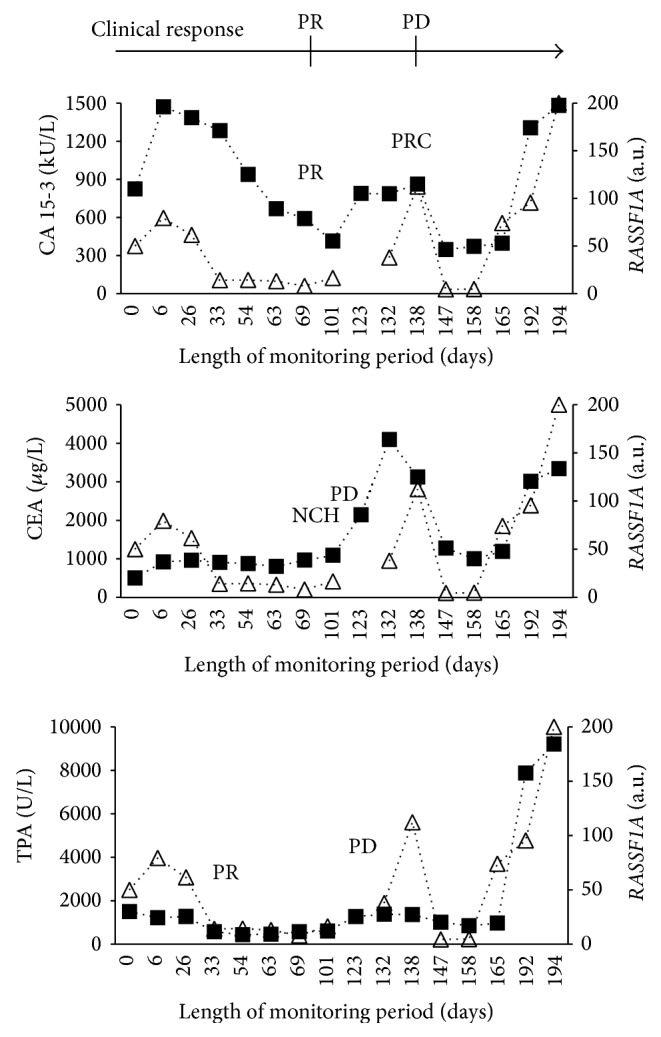
Monitoring Patient A with advanced breast cancer by measuring serial concentrations of CA 15-3, CEA, TPA, and hypermethylated* RASSF1A*. ■ denotes the respective protein biomarkers CA 15-3, CEA, and TPA. △ denotes the hypermethylated RAS association (RalGDS/AF-6) domain family member 1A gene (*RASSF1A*). The clinical response changed from a partial response (PR) to progressive disease (PD) during chemotherapy. Sixteen serial serum samples were investigated. The marker response was partial response (PR), partial response continued (PRC), no change high (NCH), and progressive disease (PD) for CA 15-3, CEA, and TPA according to previously reported assessment criteria [4].

**Figure 2:**
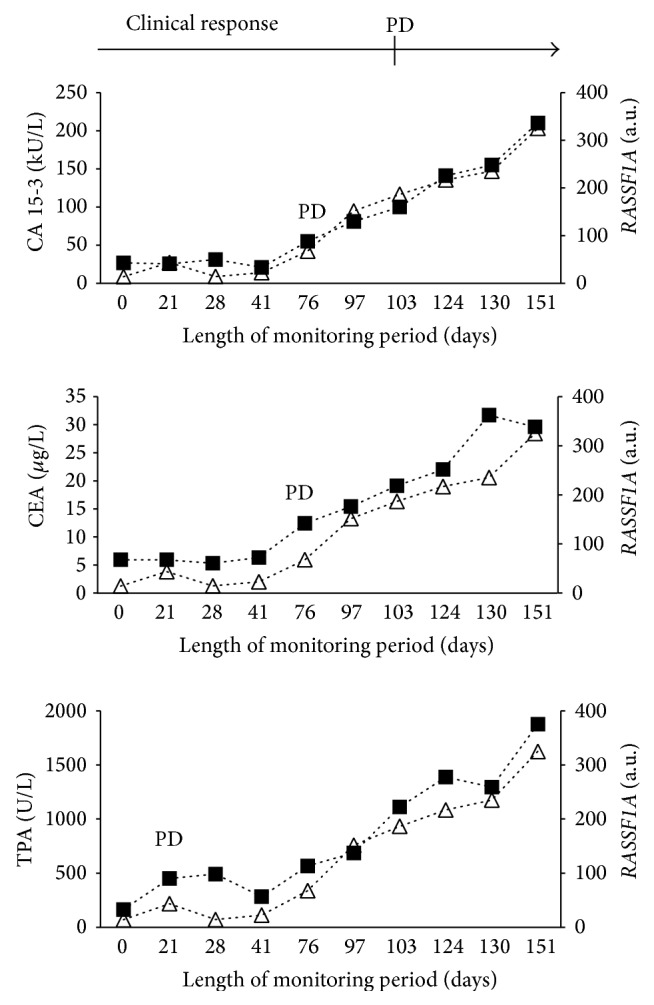
Monitoring Patient B with advanced breast cancer by measuring serial concentrations of CA 15-3, CEA, TPA, and hypermethylated* RASSF1A*. ■ denotes the respective protein biomarkers CA 15-3, CEA, and TPA. △ denotes the hypermethylated RAS association (RalGDS/AF-6) domain family member 1A gene (*RASSF1A*). The clinical response was progressive disease (PD) during chemotherapy. Ten serial serum samples were investigated. The marker response was progressive disease (PD) for CA 15-3, CEA, and TPA according to previously reported assessment criteria [4].

**Figure 3:**
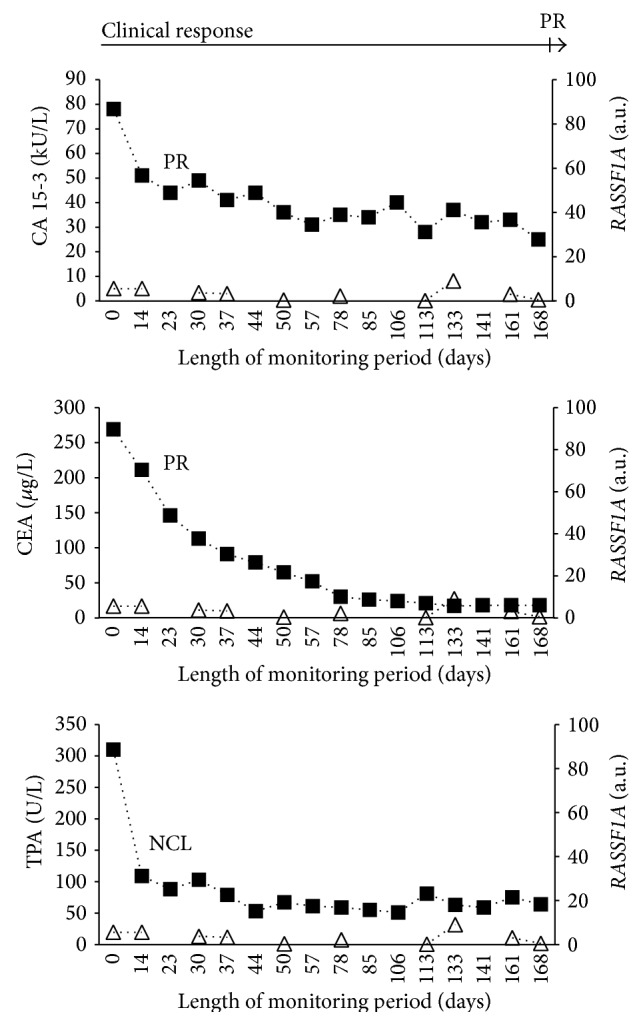
Monitoring Patient C with advanced breast cancer by measuring serial concentrations of CA 15-3, CEA, TPA, and hypermethylated* RASSF1A*. ■ denotes the respective protein biomarkers CA 15-3, CEA, and TPA. △ denotes the hypermethylated RAS association (RalGDS/AF-6) domain family member 1A gene (*RASSF1A*). The clinical response was a partial response (PR) during chemotherapy. Sixteen serial serum samples were investigated. The marker response was partial response (PR) for CA 15-3 and CEA. For TPA the response was no change low (NCL) according to previously reported assessment criteria [4].* RASSF1A* was undetectable in six samples.

**Figure 4:**
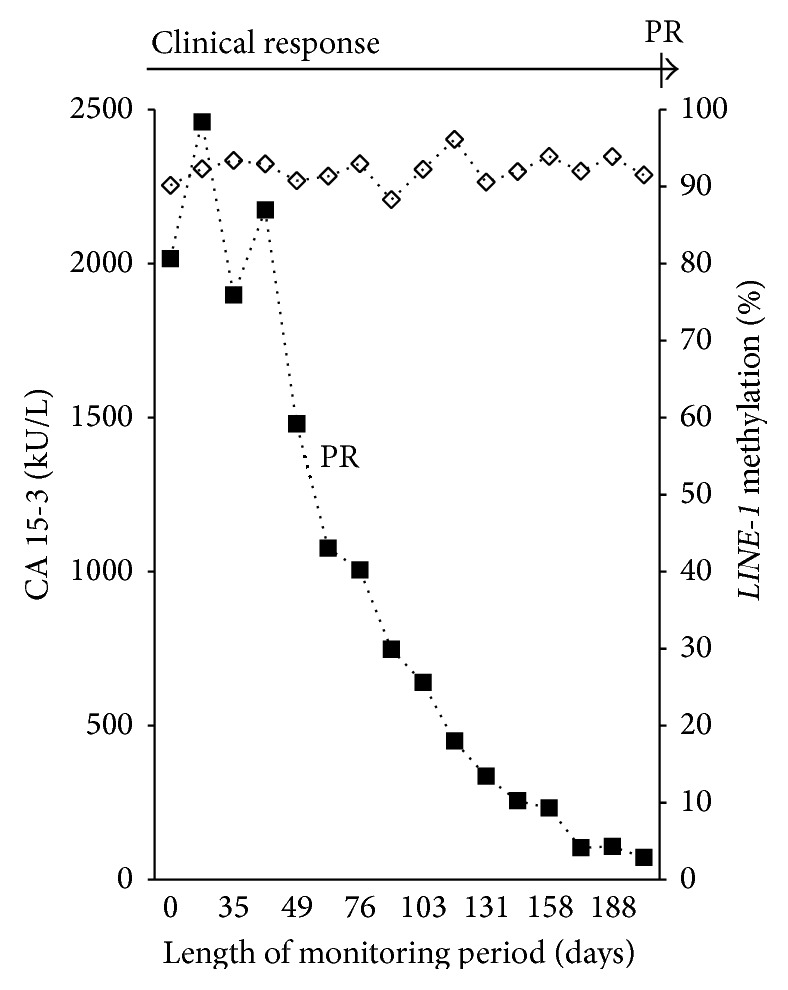
Monitoring Patient D with advanced breast cancer by measuring serial concentrations of CA 15-3 and hypomethylated* LINE-1*. ■ denotes the protein biomarker CA 15-3. ◊ denotes the hypomethylated long interspersed nuclear elements 1 (*LINE-1*). The clinical response was partial response (PR) during chemotherapy. Sixteen serial serum samples were investigated. The marker response was partial response (PR), according to previously reported assessment criteria [4].

The percentage of biomarkers with below cut of level concentrations at the start of therapy was 41.4% (CA 15-3), 69.0% (CEA), and 24.1% (TPA). The clinical evaluations and protein marker evaluations at the end of therapy are shown in Table 2. In total, 422 serial serum samples were collected from the patients during therapy with a mean of 14.5 samples per patient. Hypermethylated* RASSF1A* was detected in all of the 29 patients at some time during monitoring and was detected in 45% of the serial samples. Thus,* RASSF1A* was only periodically detected in some patients during monitoring. The monitoring data for four representative patients, Patients A-D, are provided in Figures 1–4. The interrelationship between clinical evaluations and changes in serial concentrations of the protein tumor markers CA 15-3, CEA, TPA, and* RASSF1A* in samples from Patient A appears in Figure 1. Accordingly, Patient A presents with both clinical and protein tumor marker response of PR followed by PD. The clinical PR was based on an observation of a reduction of tumor size in the contralateral mamma, bones, and lymph nodes. The clinical PD was based on increased tumor burden at several sites. There was concordance between the changes of the hypermethylated* RASSF1A* with those of CA 15-3, CEA, and TPA as well as concordance with the clinical response evaluations (PR to PD).  Figure 2 shows serial sets of data obtained from Patient B who had clinical PD during treatment as well as PD of the three protein markers. The clinical PD was based on liver and bone metastases. There was concordance between the increments in the hypermethylated* RASSF1A* concentrations with the increments of the CA 15-3, CEA, and TPA concentrations as well as concordance with the clinical response evaluations (PD). Concordance between the change in* RASSF1A* and the protein markers was also observed in Patient C who had clinical response (PR) to the treatment as shown in Figure 3. The PR evaluation was based on a reduction in bone metastases. However, there was discordance between* LINE-1* hypomethylation and the three protein tumor markers among six patients. This is illustrated for CA 15-3 and* LINE-1* for one representative patient (Patient D) who had clinical PR based on reduction of bone metastases (Figure 4).

## 4. Discussion

In the present study, concordance of changes in serum concentrations of the hypermethylated* RASSF1A* with the tumor burden markers CA 15-3 and CEA and the tumor activity marker TPA has been demonstrated for the first time. Fackler et al. [9] also monitored circulating tumor DNA in metastatic breast cancer using a 10-gene panel of hypermethylated biomarkers including* RASSF1A*. They suggested that the concentration of the methylated genes in the panel correlated with the tumor burden as evaluated by the RECIST criteria [17]. However, the change in concentrations of the investigated genes was not compared with the kinetics of CA 15-3, CEA, and TPA [9].

In some samples, we observed that hypermethylated* RASSF1A* could not be detected. One example is illustrated by Patient C (Figure 3) where* RASSF1A* remained undetected in 6 out of the 16 serial serum samples. The CA 15-3 and TPA concentrations tended to be lower as compared with the concentrations obtained for Patient A and Patient B (Figures 1 and 2, resp.). This may indicate a relatively lower tumor burden and tumor activity in Patient C and suggests why* RASSF1A* was not detected among 6 of the 16 serial samples from Patient C (Figure 3).

The hypothesis of undetectable* RASSF1A* concentrations among patients with a small tumor burden is supported by our findings among 18 healthy females where presence of* RASSF1A* in the serum samples could not be demonstrated. The findings may support the view that there is no or alternatively there is a very low release of hypermethylated* RASSF1A* into the circulation among healthy individuals and among patients with low tumor burden or low activity of the tumor(s).

It may also be speculated that the periodically lack of detection of* RASSF1A* was due to errors in preparing the serum samples for PCR analysis, that is, poor recovery of DNA and incomplete conversion of the DNA fragments during incubation with sodium bisulfite. However, this is not a likely explanation since* COL2A1* was detectable in all sequentially serum samples. We also investigated whether the periodically lack of detection of* RASSF1A* in some patients could be due to rapid degradation of the sodium-bisulfite converted DNA. Time-course analysis of* APC* (adenomatous polyposis coli gene),* CCND2* (cyclin D2 gene),* CDKN2A* (cyclin-dependent kinase inhibitor 2A gene)*, DAPK* (death-associated protein kinase 1 gene),* COL2A1*, and* RASSF1A* concentrations revealed no detectable temperature-dependent degradation of the bisulfite-converted DNA when stored for one day, 7 days, 30 days, and 60 days at 4°C, −20°C and −80°C (data not shown). Finally, thawing and immediately refreezing at −20°C 10 times did not result in any detectable change in the* COL2A1* concentration (data not shown). Taken together, the stability study showed that the sodium bisulfite-converted DNA was stable, and the periodically lack of* RASSF1A* detection in some patients may be explained by* in situ* subdetectable concentrations.

In conclusion, circulating hypermethylated* RASSF1A* and protein cancer biomarkers may have similar kinetics during monitoring of tumor burden among patients with advanced breast cancer. However, further investigations are needed to determine whether any of the hypermethylated DNA genes may provide predictive information during monitoring.

## Figures and Tables

**Table 1:** Distribution of metastasis before and after therapy among the 29 investigated patients.

	Start of therapy	End of therapy
Lung	31.0% (9/29)	24.1% (7/29)
Liver	3.4% (1/29)	13.8% (4/29)
Contralateral mamma	3.4% (1/29)	0.0% (0/29)
Bone	44.8% (13/29)	48.3% (14/29)
Intra-abdominal	3.4% (1/29)	3.4% (1/29)
Skin	20.7% (6/29)	10.3% (3/29)
Lymph node	48.3% (14/29)	20.7% (6/29)
Other locations	13.8% (4/29)	17.2% (5/29)
CNS	n.d.	3.4% (1/29)
Solitary location	20.7% (6/29)	6.9% (2/29)
Multiple location	75.9% (22/29)	69.0% (20/29)

**Table 2:** Responses based on clinical evaluations and protein marker evaluations at the end of therapy among the 29 patients.

Evaluation	Complete response (CR)	Partial response (PR)	No change (NC)	Progressive disease (PD)
Clinical evaluation	24% (7/29)	14% (4/29)	31% (9/29)	31% (9/29)
CA 15-3 evaluation	14% (4/29)	24% (7/29)	48% (14/29)	14% (4/29)
CEA evaluation	10% (3/29)	17% (5/29)	59% (17/29)	14% (4/29)
TPA evaluation	14% (4/29)	7% (2/29)	55% (16/29)	24% (7/29)

## References

[1] National Comprehensive Cancer Network (NCCN) (2015). *Clinical Practice Guidelines in Oncology. Breast Cancer. Version 3*.

[2] Sölétormos G. (2001). Serological tumor markers for monitoring breast cancer. *Danish Medical Bulletin*.

[3] Diamandis E. P., Fritsche H. A., Lilja H., Chan D. W., Schwartz M. K. (2002). *Tumor Markers: Physiology, Pathobiology, Technology and Clinical Applications*.

[4] Sölétormos G., Nielsen D., Schiøler V., Skovsgaard T., Dombernowsky P. (1996). Tumor markers cancer antigen 15.3, carcinoembryonic antigen, and tissue polypeptide antigen for monitoring metastatic breast cancer during first-line chemotherapy and follow-up. *Clinical Chemistry*.

[5] Fernandez A. F., Assenov Y., Martin-Subero J. I. (2012). A DNA methylation fingerprint of 1628 human samples. *Genome Research*.

[6] Kristiansen S., Jørgensen L. M., Guldberg P., Sölétormos G. (2013). Aberrantly methylated DNA as a biomarker in breast cancer. *International Journal of Biological Markers*.

[7] Kristiansen S., Nielsen D., Sölétormos G. (2014). Methylated DNA for monitoring tumor growth and regression: how do we get there?. *Critical Reviews in Clinical Laboratory Sciences*.

[8] Heichman K. A., Warren J. D. (2012). DNA methylation biomarkers and their utility for solid cancer diagnostics. *Clinical Chemistry and Laboratory Medicine*.

[9] Fackler M. J., Bujanda Z. L., Umbricht C. (2014). Novel methylated biomarkers and a robust assay to detect circulating tumor dna in metastatic breast cancer. *Cancer Research*.

[10] Sunami E., Vu A.-T., Nguyen S. L., Giuliano A. E., Hoon D. S. B. (2008). Quantification of LINE1 in circulating DNA as a molecular biomarker of breast cancer. *Annals of the New York Academy of Sciences*.

[11] Soletormos G., Schioler V., Nielsen D., Skovsgaard T., Dombernowsky P. (1993). Interpretation of results for tumor markers on the basis of analytical imprecision and biological variation. *Clinical Chemistry*.

[12] Nielsen D., Dombernowsky P., Larsen S. K., Hansen O. P., Skovsgaard T. (2000). Epirubicin or epirubicin and cisplatin as first-line therapy in advanced breast cancer. A phase III study. *Cancer Chemotherapy and Pharmacology*.

[13] World Health Organization (1979). *WHO Handbook for Reporting Results of Cancer Treatment*.

[14] Friedrich M. G., Weisenberger D. J., Cheng J. C. (2004). Detection of methylated apoptosis-associated genes in urine sediments of bladder cancer patients. *Clinical Cancer Research*.

[15] Müller H. M., Widschwendter A., Fiegl H. (2003). DNA methylation in serum of breast cancer patients: An independent prognostic marker. *Cancer Research*.

[16] Iacopetta B., Grieu F., Phillips M. (2007). Methylation levels of LINE-1 repeats and CpG island loci are inversely related in normal colonic mucosa. *Cancer Science*.

[17] Eisenhauer E. A., Therasse P., Bogaerts J. (2009). New response evaluation criteria in solid tumours: revised RECIST guideline (version 1). *European Journal of Cancer*.

